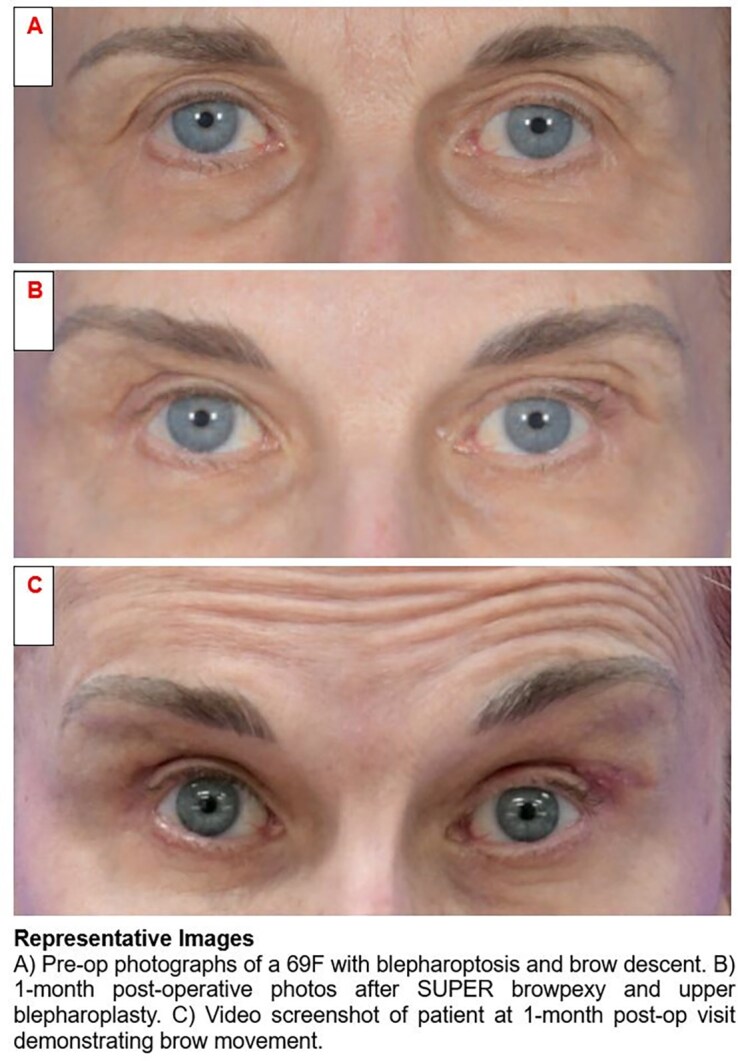# The SUPER Browpexy, or the Scarless Upper Periosteal-Flap Eyebrow Restoring Browpexy: A Technique That Maintains Brow Movement Via a Superiorly-Based Periosteal Flap

**DOI:** 10.1093/asjof/ojaf018.010

**Published:** 2025-05-13

**Authors:** Alexandra Lin, Amanda Young, David Staffenberg

**Affiliations:** NYU Langone Health, New York, NY; NYU Langone Health, New York, NY; NYU Langone Health, New York, NY

## Abstract

**Goals/Purpose:**

Lateral descent of the brow is one of the earliest signs of aging. From a functional standpoint, as the soft tissue of the brow descends over the supraorbital rim, patients may develop eyelid ptosis, leading to visual impairment. From an aesthetic standpoint, drooping lateral brows create the appearance of someone who is sad or fatigued.

The goal or any browlift or browpexy procedure is to restore the youthful position of the brow. The peak of the brow is ideally located above the lateral limbus of the pupil and is a key factor in determining aesthetic shape. In women, this peak should be located several millimeters above the supraorbital rim while in men it should be around the level of the rim.

Brow rejuvenation procedures are challenging in that overcorrecting a drooping lateral brow gives patients a startled or surprised look. At the same time, undercorrection with recurrence of brow ptosis is an undesirable result for both patient and surgeon. Functionally, an ideal aesthetic brow procedure should also account for brow movement, as eyebrow shape and position contribute significantly to facial expression.

Our aim is to describe a transblepharoplasty browpexy technique that allows for normal brow movement while additionally being straightforward and effective.

**Methods/Technique:**

The ideal patient for the SUPER browpexy has the following characteristics:

1. A short or normal forehead length without a receding hairline
2. Maintenance of medial brow position with any degree of lateral and central brow ptosis
3. Indicated for an upper blepharoplasty procedure

Patients with a longer forehead who are extremely averse to the idea of pretrichial or direct brow lift scars may also be candidates as long as they understand that this technique will not address excess skin of the forehead.

The technique begins with a standard upper blepharoplasty incision and skin excision, taking care to account for final brow position. The supraorbital rim is approached as one would do for standard transblepharoplasty browpexy. Once the periosteum is exposed, a 10-12mm wide, superiorly-based periosteal flap is raised from the supraorbital rim to the planned final position of the arc of the brow (at least 5mm above the orbital rim with additional 1-2mm for overcorrection). The peak of the eyebrow is then sutured to the superior extent of this periosteal flap, taking care to incorporate the orbicularis/frontalis muscle complex and exclude the dermis in this location. This pexy relocates the arc of the brow to its new position on a relatively mobile periosteal flap. Hemostasis is rechecked and the upper lid is then closed in a standard fashion.

**Results/Complications:**

The SUPER browpexy technique presents several benefits for both surgeon and patient.

For the surgeon:

1. It is anatomically straightforward with limited risk for damage to nearby neurovascular structures.
2. Minimal dissection means adding very little time to the case (approximately <10 minutes per side in our experience).
3. It requires no special equipment or fixation material.

For the patient:

1. Using the upper blepharoplasty incision means that it is a “scarless” procedure.
2. Limited dissection leads to less bruising and a faster return to normal activity.
3. Fixation of the orbicularis/frontalis complex to a mobile periosteal flap means that patients are able to maintain normal movement of their eyebrows, allowing for more natural expressions.

Limitations are similar to other techniques for browlifting and can include recurrence of brow ptosis and slight asymmetry.

**Conclusion:**

We present one of our preferred techniques for elevation of the lateral brow via fixation of the orbicularis oculi/frontalis muscle complex to a periosteal flap to rejuvenate the arc of the brow. As it can be performed via an upper blepharoplasty incision, it is additionally “scarless,” making it an excellent choice for the demanding patient.

**Figure:**